# Controlling Kinetic Pathways in Demixing Microgel–Micelle Mixtures

**DOI:** 10.1021/acs.langmuir.2c02583

**Published:** 2023-01-09

**Authors:** S. L. Fussell, C. P. Royall, J. S. van Duijneveldt

**Affiliations:** † School of Chemistry, University of Bristol, Cantock’s Close, Bristol BS8 1TS, U.K.; ‡ Bristol Centre for Functional Nanomaterials, University of Bristol, Tyndall Avenue, Bristol BS8 1TL, U.K.; § Gulliver UMR CNRS 7083, ESPCI Paris, Université PSL, 75005 Paris, France; ∥ HH Wills Physics Laboratory, University of Bristol, Tyndall Avenue, Bristol BS8 1TL, U.K.

## Abstract

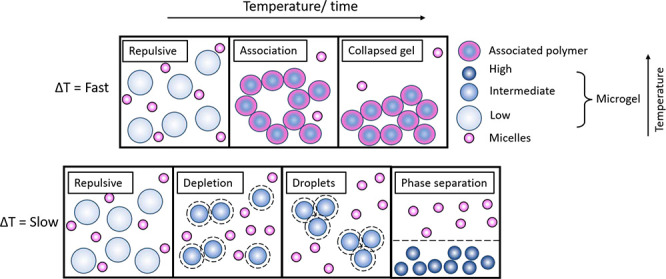

We investigate the temperature-dependent phase behavior of mixtures of poly(*N*-isopropylacrylamide) (pNIPAM) microgel colloids and a triblock copolymer (PEO–PPO–PEO) surfactant. Usually, gelation in these systems results from an *increase* in temperature. Here we investigate the role of the heating rate, and surprisingly, we find that this causes the mechanism of aggregation to change from one which is driven by depletion of the microgels by the micelles at low temperatures to the association of the two species at high temperatures. We thus reveal two competing mechanisms for attractions between the microgel particles which can be controlled by changing the heating rate. We use this heating-rate-dependent response of the system to access multiple structures for the same system composition. Samples were found to demix into phases rich and poor in microgel particles at temperatures below 33 °C, under conditions where the microgels particles are partially swollen. Under rapid heating full demixing is bypassed, and gel networks are formed instead. The temperature history of the sample, therefore, allows for kinetic selection between different final structures, which may be metastable.

## Introduction

While much research is performed at room temperature, one may enquire as to the response of soft materials to changes in temperature. These are often complex. In the case of colloid–polymer mixtures, for example, when attractions between the colloids are driven by polymer-induced depletion, heating has surprising consequences: upon heating, polymer molecules can expand, thus increasing the polymer volume fraction and hence effectively quenching the system by increasing the attraction between the colloids. In this, one may observe the counterintuitive behavior of (effectively) quenching by heating and raising the effective temperature by cooling.^1^ Often more complex behavior can be observed due, for example, to changes in hydrophobicity as a function of temperature between different species in the system. The system of interest here is a mixture of microgel particles and triblock copolymer micelles, which exhibits a complex response to temperature, in addition to a composition-dependent phase behavior typical of soft materials.^2−7^

In the case that the interactions are controlled through the composition of the system (rather than temperature), the attractions that drive phase separation can have a variety of sources, including the depletion interaction between the (microgel) colloids induced by *nonabsorbing* polymers noted above,^8,9^*bridging* between colloidal particles by the polymers,^10,11^ or through the critical Casimir effect.^12,13^ Under the right conditions, such attractions can lead to a network of colloids formed through spinodal decomposition, which undergoes dynamic arrest due to the asymmetry in viscosity of the two demixing phases, which is termed viscoelastic phase separation, or sometimes no attractions are actually needed to form such a network of fluids with very different viscosities.^14^ Whatever the origins of the attractions which lead to the emergence of the viscoelastic network, *understanding* colloidal gelation due to arrested spinodal decomposition is a long-standing challenge,^9,15−20^ although the mechanism of solidification has been related to the emergence of rigid clusters, leading to a gel with viscoelastic mechanical properties.^21,22^

Gels are far-from-equilibrium systems, and their properties exhibit complex time-dependent behavior, as they relax toward the equilibrium state of phase coexistence between the colloid-rich (high-viscosity) phase and colloid-poor (low viscosity) phase.^17,20,22−30^ Numerical studies of the assembly of so-called “sticky spheres” out of equilibrium, which is a basic model system which undergoes gelation where the colloid-rich phase may crystallize,^31^ have shown that assembly at different effective temperatures (expressed via the inverse of the strength of the effective attraction between the particles) at different temperatures leads to a complex response of fast yet poor-quality assembly upon deep (effective) quenches and slower but high-quality assembly when the (effective) temperature is higher.^32^ This is backed up to some extent by experimental work where the system evolved more quickly to its equilibrium demixed state in the case of weak quenches.^29,33^ Time-dependent assembly protocols have also been investigated in computer simulations, and it has been found that slow quenches promote high-quality assembly,^24^ and time-dependent protocols of a deep quench at short times followed by a shallow quench at longer times optimize the process of assembly further.^34^ While these numerical studies have given stimulus and ingenious experimental systems provide the means to access time-dependent assembly protocols,^35^ the study of the temperature dependence of assembly protocols in colloidal systems is in its infancy, and this we address here. We consider a soft matter system that displays both phase separation to a colloidal gas–liquid phase coexistence (liquid–liquid phase separation) and formation of gel networks. Which path is chosen depends on the heating rate. Such systems that are capable of triggered gelation or viscosity-switchable materials are sought after for reconstructive surgery and drug delivery applications.^36^

This paper is organized as follows: We first describe our model system in more detail. We then present our methods, followed by our main findings including the phase behavior, control over the state of the system using quench rates, and detailed characteristics of our system.

## A Model Microgel–Micelle System

Our system is based on a mixture of pNIPAM microgels and triblock copolymer surfactant micelles. The micelles are formed from a PEO–PPO–PEO triblock copolymer.^37^ Microgels are colloidal particles composed of a cross-linked polymer^38−43^ which in some ways behave similarly to hard colloids.^42,44,45^ Microgels are know to have a complex phase behavior and response to temperature. In particular, poly(*N*-isopropylacrylamide) (pNIPAM) microgels are known to form colloidal gels in the presence of additives, such as salt, which screens the electrostatic interactions.^46−48^ Their swelling behavior is temperature-responsive, which is a result of the polymer–solvent interactions.^49^

Detailed phase diagrams have been built describing the temperature-dependent behavior of pNIPAM microgels.^48,50^ Complex behavior is found not only with increased temperature but with variable quench rates. For example, in mixed microgel systems, fine control over the gel network formed can be achieved by varying the quench rate, where bigels are formed on slow heating of binary microgel mixtures, where each microgel has a unique volume phase transition temperature (*T*_VPT_).^47^

**Figure 1:**
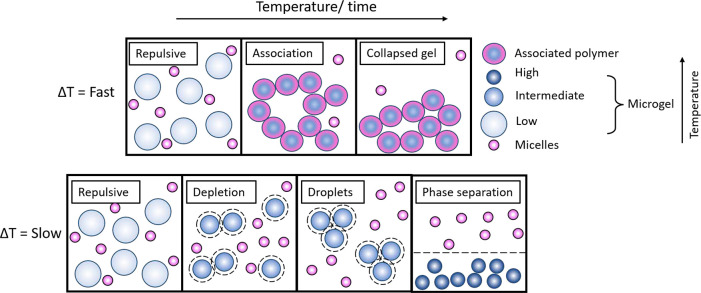
Schematic illustrating the behavior of pNIPAM microgels in the presence of triblock copolymer, contrasting slow and fast heating rates. The interactions are shown as a function of both temperature and time. The top layer shows samples that are heated quickly. At low temperatures the microgel particles are repulsive; then when heated quickly, they associate strongly, likely through the micelles/surfactant adsorbing inside the microgels preventing them from deswelling. This results in a particle network without the ability to rearrange, resulting in the formation of collapsing gels. The bottom layer highlights the behavior when samples are heated slowly. At low temperatures the samples are again repulsive; then depletion occurs at intermediate temperatures when the pNIPAM is still swollen. This results initially in the formation of droplets of colloidal liquid, which are rich in microgel particles, and eventually in phase separation.

Our model system is a mixture of microgels and micelle-forming triblock copolymers which exhibits a complex response to temperature.^2^ Upon heating, there are three underlying effects: First, the microgels undergo the volume phase transition and collapse over a temperature range of around 10 °C, between 30 and 40 °C.^51,52^ The second effect, which drives the gelation in our system,^2^ is that upon heating above the *T*_VPT_, the triblock copolymers associate with the microgels due to hydrophobic interactions, which leads to the microgel particles aggregating and undergoing gelation (Figure 1, top scheme). Thus, here, the system is *effectively* quenched by *increasing temperature*,^2^ in a manner reminiscent of the mixtures of colloids and nonabsorbing polymers noted above.^1^ The third effect is that at intermediate temperatures between room temperature and the *T*_VPT_, depletion drives the aggregation in our systems. Here, we explore the response of our system to *time-dependent temperature protocols*. We find a complex behavior resulting from the competition between thermodynamic forces driving phase separation and slow dynamics. By changing the *effective* quench rate, we can tune the system toward arrested phase separation, when the rate of temperature change is fast with respect to the demixing kinetics or toward phase separation when the rate is slow with respect to demixing kinetics.

We use differential interference contrast and confocal microscopy to investigate the structure of the gel networks, and study the phase behavior of this system. First, we observed the high temperature gelation of these species, but also a change in the phase behavior, as a result of altering the conditions by which gelation occurred. Second, under specific conditions, we observe demixing (phase separation), where microgel rich and poor liquid regions form. We highlight the trends in phase behavior that results from tuning the gelation conditions (quench rate) and the sample concentration.

## Experimental Section

### Materials

For the microgel synthesis *N*,*N*′-methylenebis(acrylamide) (99%, Sigma-Aldrich), *N*-isopropylacrylamide (99%, Acros Organics), potassium persulfate (99%, Sigma-Aldrich), sodium dodecylbenzenesulfonate (technical grade, Sigma-Aldrich), fluorescein isothiocyanate (98%, Sigma-Aldrich), and methacryloxyethyl thiocarbonyl rhodamine B (Polysciences) were used as received without further purification. The triblock copolymer was labeled using Nile red dye (technical grade, Sigma-Aldrich). Synperonic SE/P105 is the triblock copolymer used for all the experiments, a poly(ethylene oxide)–poly(propylene oxide)–poly(ethylene oxide) (PEO–PPO–PEO) type polymer (6500 MW).

### PNIPAM Microgel Synthesis

The microgels were synthesized using precipitation polymerization.^52^ Each synthesis was conducted in a 1000 mL three-necked round-bottom flask, fitted with a condenser, an argon inlet, and an overhead stirrer. NIPAM (12.5 g), *N*,*N*′-methylenebis(acrylamide) (1.0 g), and sodium dodecyl benzenesulfonate (0.5 g) were added to the flask along with 475 mL of deionized water and heated to 70 °C with stirring under an inert atmosphere. Once heated, potassium persulfate (0.5 g dissolved in 25 mL of DI water) was slowly added to the reaction, and the solution was left stirring at temperature for 4 h. The microgels produced were then purified using dialysis against deionized water for 2 weeks. Fluorescein-labeled particles were synthesized using 9 mg of fluorescein isothiocyanate and 15 μL of 3-aminopropene and added to 38 mL of a solution of 10^–4^ M sodium hydroxide. This solution was then added at the start of the pNIPAM reaction discussed above, in place of 38 mL of water.^53^ All particles were then characterized using a Malvern Autosizer 4800 with a 532 nm laser at a 90° scattering angle. This was used to determine the diameter of the particles and to study the deswelling behavior. All samples were diluted using deionized water and left to equilibrate at each temperature for 15 min.

### Microscopy

An Olympus BX-51 DIC microscope was used to obtain the differential interference contrast (DIC) images. All images were captured using a Pixelink 5MP color CCD PL-B625CU camera and a 20× objective. A Linkam PE120 heating stage was used to control the slide temperature. For the fast heating experiments, the samples were heated at a rate of 10 °C min^–1^ from room temperature to 35 °C. For the slow heating experiments, the samples were heated at a rate of 0.1 °C min^–1^ to 35 °C unless otherwise stated. The DIC images have been edited using ImageJ software. The images have been converted to gray scale, then to the “glow” color table, and then the image inverted. This was done to highlight the polymer-rich phase using this imaging technique. An example of the process to edit the DIC images is included in the Supporting Information (Figure S1).

Confocal images were taken using a Leica SP5 confocal microscope. The sample preparation was as described for DIC microscopy along with the heating protocol. The micelles were labeled with Nile red; a 543 nm HeNe laser was used to excite the dye. The microgels were labeled with fluorescein, and an argon laser (488 nm) was used to excite the dye. All samples were heated using a TC-1-100s temperature controller, along with a Bioscience Tools objective heater, set to 35 °C.

### Macroscopic Characterization of Phase Behavior

To replicate the fast heating experiments for macroscopic gels, the samples were made up in sealed vials and submerged in hot water (approximately 70 °C). The phase behavior was then recorded after 10 min. The temperatures was higher than the microscopic quench due to the slower rate of heating the larger microscopic samples and to reduce edge effects. To replicate the slow heating experiments, samples were placed into an oven set to 40 °C and allowed to slowly heat up from room temperature, and the phase behavior was recorded after 24 h. The temperatures were different to the microscopic samples.

### Dynamic Light Scattering (DLS)

The microgel particles and triblock copolymer micelles were characterized using DLS. The data were collected using a Malvern Autosizer 4800 at a 90° scattering angle with a 532 nm laser. Samples were characterized at multiple temperatures, with each held at the desired temperature for 15 min to allow for equilibration to occur. For fast heating, samples were submerged into a water bath at 50 °C before being placed in the DLS.

## Results and Discussion

In this section, we present the temperature-induced gelation of pNIPAM microgels and triblock copolymer micelles. We present the temperature response of these systems characterized at both the macroscopic (cm) and microscopic (μm) length scale. We characterize the structure formed in these mixtures using DIC and confocal microscopy. We also use DIC imaging to characterize the temperature where the microgel particles aggregate (Figure 4), and highlight how the temperature response at the particle level can be related to the structures observed. We alter the heating rate in order to determine the relationship between the heating rate and the structures observed. We use confocal microscopy to identify the position of microgel and micelle species in the structures formed.

### Effect of Heating Rate

#### Fast Heating: Gelation by Association

We begin our presentation of our results by considering the case of fast heating (10 °C min^–1^ from room temperature to 35 °C), a temperature above the *T*_VPT_ for pNIPAM, meaning the microgels used in this study are close to fully deswollen (Figure 3). Samples of pNIPAM microgels and triblock copolymer micelles were heated and imaged using DIC microscopy. Upon heating at a rate of 10 °C min^–1^, there is very little observable difference in the gel structures, regardless of the concentration of *either* of the two species: microgel colloids or micelles. The structure observed for all samples is a uniform gel network. The gel branches are of order 1 μm width with very few observable pores (Figure 5). The mechanism for gelation upon fast heating is one of association, where the microgels and micelles associate due to hydrophobic interactions and hydrogen bonding (Figure 1). This association was characterized previously, using small-angle neutron scattering, confocal microscopy, and DLS.^2^

The concentrations investigated here were 1–5 wt % pNIPAM microgels and 1–10 wt % triblock copolymer micelles. The lower concentrations of microgels and micelles show evidence of aggregates forming with heating, as the samples become increasingly opaque compared to microgels alone. This was also evidenced using DLS, where above the *T*_VPT_ aggregates larger than the individual microgel particles are observed (Figure 3).

At high enough concentrations of the micelles and microgels, the mixtures form macroscopic gels (>3 wt % microgel), which are originally space spanning and subsequently collapse over time, This behavior has been investigated in our previous work.^2^ The shrinking behavior is very similar to that observed by Trappe et al.,^54^ where they saw a decrease in volume of up to 90% for their collapsing gels consisting of pNIPAM microgels in the presence of phosphate buffer solution. They report a new property of pNIPAM microgels not extensively discussed in the literature, being a second significant temperature above the *T*_VPT_, where the pNIPAM particles are fully collapsed; in their work this was found to be 36 °C. Between the *T*_VPT_ and this temperature the particles continue to contract with increased temperature. This was also observed as part of this system, where we observe an interaction using DLS only at temperatures the particles are fully collapsed (Figure 3).

**Figure 2:**
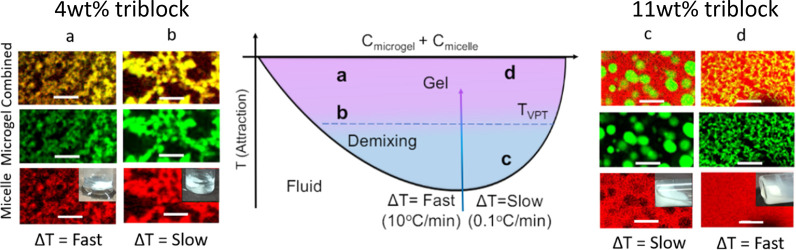
Scheme representing the observed behavior of samples of pNIPAM and triblock copolymer, imaged using confocal microscopy. For a sample heated slowly, phase separation is observed (blue) at lower temperatures and gelation (pink) at higher temperatures. If the sample is heated quickly, the liquid–liquid phase separation is bypassed, and only gels (pink) are observed at all concentrations. All samples contain 3 wt % pNIPAM; the images on the left are of samples with 4 wt % triblock copolymer. The images on the right are of samples with 11 wt % triblock copolymer. Images in columns b and c have been heated slowly in an oven set to 40 °C. Images in columns a and d are of samples that have been heated quickly in a water bath. The green images are of the labeled microgels, the red images are of the labeled micelles, and the combined image of both dyes is also included. The scale bar included for all the confocal images is 10 μm.

Confocal microscopy indicates that both the microgels and micelles are present in the gel network, where the green fluorescent signal (microgels) and red fluorescent signal (micelles) can be seen in the same location of the gel, therefore indicating that association has occurred (Figure 2a,d).

#### Slow Heating: Phase Separation by Depletion

**Figure 3:**
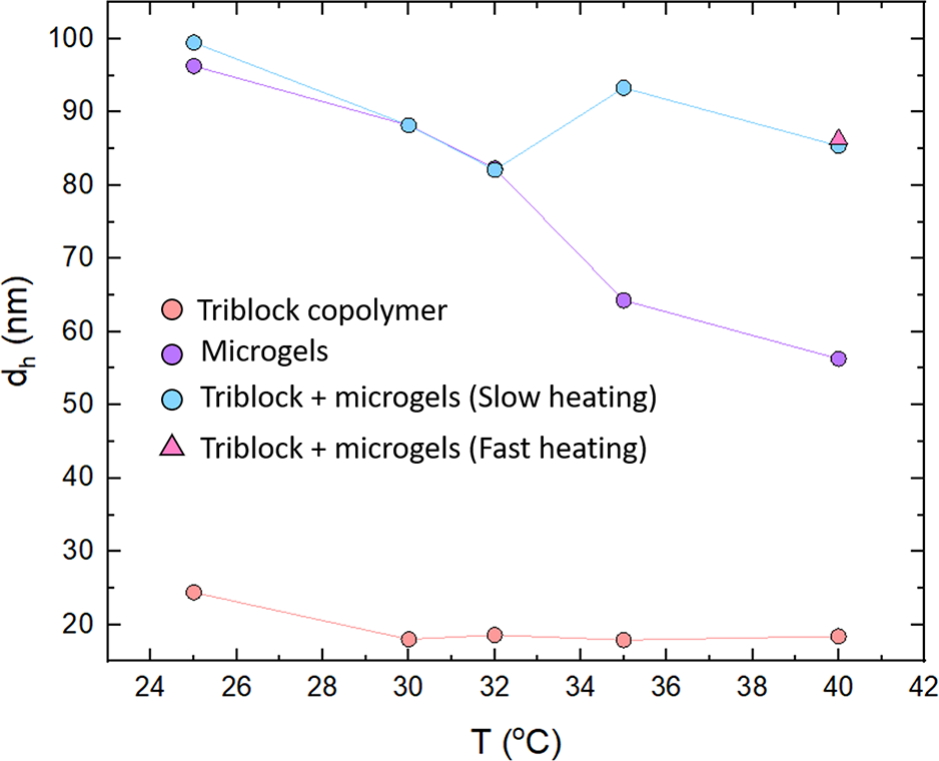
Dynamic light scattering data for the pNIPAM microgels (purple) and the triblock copolymer surfactant (peach) used in this work at different temperatures. DLS data are also included for mixtures of pNIPAM (0.15 wt %) and triblock copolymer (0.15 wt %) after slow heating (blue) and fast heating (pink).

Figure 2 summarizes the general behavior observed for mixtures of microgels and micelles when samples are heated slowly (0.1 °C min^–1^), using DIC microscopy and confocal microscopy. At high concentrations of micelles and microgels and temperatures below *T*_VPT_, demixing occurred at a micelle concentration of around 7 wt %. Droplets rich in microgels are seen. These droplets contain just the microgel species, with the micelles remaining free in solution. This was evidenced using confocal microscopy (Figure 2c). This implies the mechanism for microgel aggregation in this system is one of depletion, where nonadsorbing polymer causes colloidal aggregation. With increasing time or concentration of microgels and micelles, these droplets coalesce leading to phase separation (Figures S4–S6). This was also evidenced using DLS, where there is no evidence of the micelles and micelles associating below the *T*_VPT_ (Figure 3), indicating depletion is the likely mechanism for aggregation at these temperatures.

When mixtures of microgels and micelles are heated quickly (10 °C min^–1^), the phase separation is bypassed and colloidal gels are observed at all concentrations (Figure 2b,d). Whether phase separation or gelation occurs can be related to the quench rate in other colloidal systems. Slow quenching often results in phase separation rather than gelation.^24,55^ Gels are nonequilibrium structures, and therefore the effects of kinetic trapping can be important.^9,15−18^ Hence, in our system, gel networks are always observed on fast heating (10 °C min^–1^) as the equilibrium structure cannot be accessed. For the lower concentration samples, heating rate has little effect on the structure, and gel phases are observed at both heating rates (Figure 2a,b, 4 wt % micelle). However, for the higher concentration samples, large differences in the structure were observed (Figure 2c,d, 11 wt % micelle). This is where the aggregation of the microgels occurs at lower temperatures.

In the case of slow heating, the macroscopic observation of the phase behavior was consistent with the microscopic observations discussed above. When samples at the same composition were heated quickly, by immersion in hot water, all samples formed collapsing gels (Figure 2a,d). When the samples were left in an oven to heat slowly from room temperature to 40 °C, higher concentration samples instead showed evidence of *phase separation* rather than gelation. A layer rich in pNIPAM formed along with a layer of clear liquid (Figure 2c). These samples flowed upon inversion of the vial, highlighting that the system forms a weaker structure than the gel networks.

### Aggregation Behavior under Slow Heating

**Figure 4:**
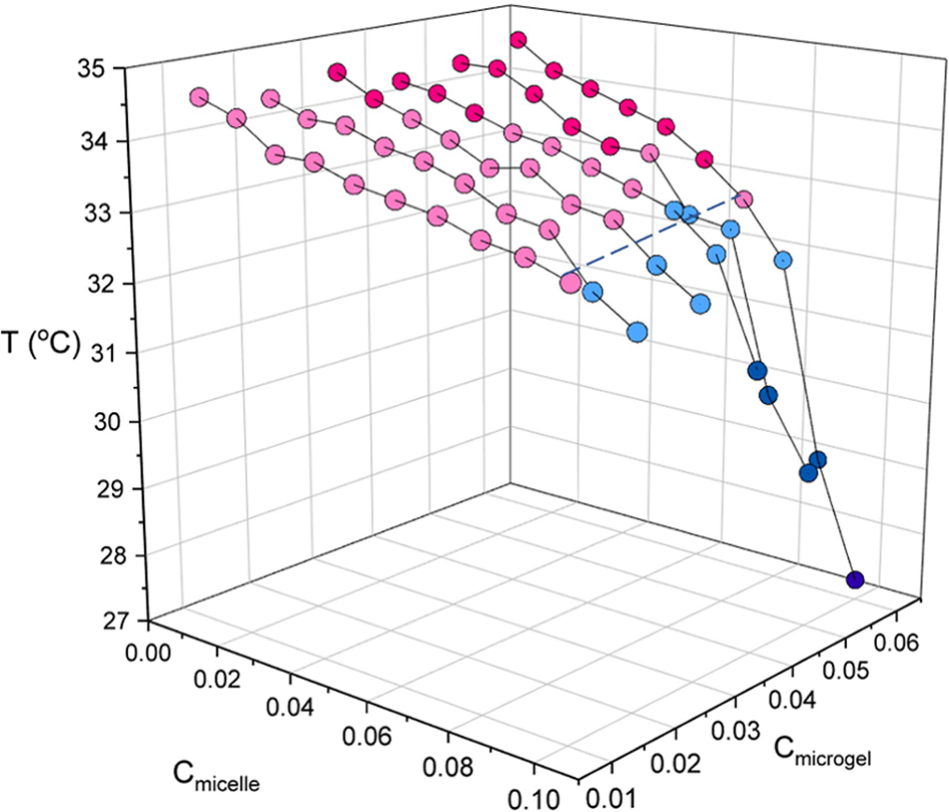
Aggregation temperatures observed using DIC microscopy. The samples were heated at a rate of 0.1 °C min^–1^ (slow), and the temperature was recorded as the point where aggregation was first observed. The colors represent the phase observed on the microscope. If gelation occurs at temperatures above about 33 °C, then gels are always observed. The dark pink circles indicate samples that are uniform gels, the light pink circles samples that form gels with voids, light blue circles samples that form droplets, and dark blue circles samples that phase separate. The dashed line highlights the temperature boundary between gelation and phase separation.

In the case of the slow heating rate, we now consider the phase boundary between fluid (low temperature) and gel or demixed phases at higher temperature as a function of concentration of microgels and triblock copolymer micelles (Figure 4, dashed line). We find changes in the nature of the demixing can be described as a function of composition as well as heating rate, as discussed above. At temperatures below approximately 33 °C droplet formation and phase separation occurred (Figure 4, blue data points), whereas at higher temperatures gels are formed (Figure 4, pink data points).

For samples undergoing demixing, the aggregation occurs below the *T*_VPT_ for a range of polymer concentrations (Figure 4). This shows that aggregation in these systems occurs when the particles are still swollen. When gels result (Figure 4, pink), aggregation occurs when the pNIPAM is close to being fully deswollen. However, for the concentrations where the aggregation temperature is lower than the *T*_VPT_, a different phase behavior is observed and phase separation occurs. The confocal images indicate a change in interaction mechanism with temperature. At lower temperatures, the mechanism for aggregation is depletion, where the micelles behave like nonadsorbing polymers resulting in aggregation, evidenced by the micelle signal remaining in the supernatant around the droplets. At higher temperatures than the *T*_VPT_, gels result, and the fluorescent signals of the micelles and microgels overlap.

Figure 4 shows a 3D representation of the effect of both the micelle and microgel concentration on the temperature of the phase boundary for aggregation. The temperature of the phase boundary decreases with increasing microgel concentration. At a low concentration of microgels (1 wt %), we encounter only gelation. Increasing the concentration of microgels to 6 wt %, we find phase separation at a progressively lower micelle concentration. Also at a low concentration of microgels, the phase boundary is almost flat as a function of micelle concentration but slopes down progressively upon increasing the microgel concentration.

**Figure 5:**
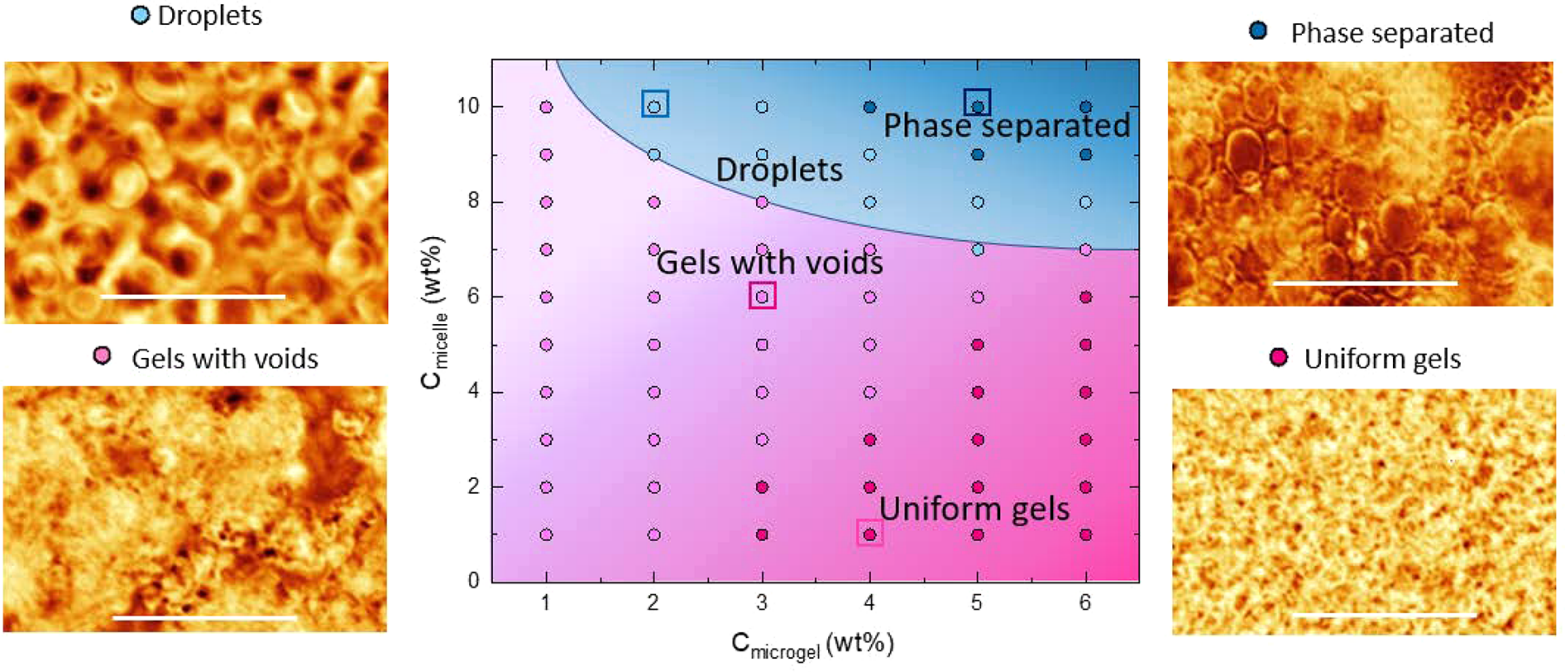
Phase behavior of mixtures of pNIPAM microgels and triblock copolymer in the *c*_microgel_–*c*_micelle_ plane, imaged using DIC microscopy. All samples were heated to 35 °C at a heating rate of 0.1 °C min^–1^ before the final phase was recorded. Pink circles indicate state points at which gel networks were observed. Light pink circles indicate where gels with larger voids were observed, and dark pink circles where uniform gels are observed. Blue circles indicate where phase separation is observed. The light blue circles indicate where isolated droplets were observed, and the dark blue circles indicate where phase separation occurred. The colored squares indicate the concentrations of the samples in the example DIC images included. The shading has been added to guide the eye. The scale bar on all the images is 40 μm.

Figure 5 details the phase behavior observed, when the samples were heated slowly (0.1 °C min^–1^). For low concentrations of micelle and microgel, the system undergoes gelation. Two types of networks were observed. The first was uniform gels, where little structural detail was observed at the resolution of the DIC microscope used (Figure 5, dark pink). These were formed at a high concentration of microgels, more than 3 wt %. The second morphology we term gels with voids, where when imaged under the DIC microscope the arms of the gel network were resolvable (Figure 5, light pink). At the lower concentrations of microgel (<3 wt %), gels with voids preferentially form, as the low volume fraction of microgel in these samples results in sparse networks. These low concentration samples macroscopically do not form gels but only show evidence of aggregates that sediment.^2^

Turning to the behavior of the system as a function of micelle concentration, we find that gels with voids also form preferentially with increasing micelle concentration. This is most prevalent at concentrations close to the boundary where phase separation is observed. To explain this trend, we looked in more detail at the aggregation temperature as a function of both micelle and microgel concentration. This is summarized in Figure 4. It can be seen that the aggregation temperature decreases with increasing micelle concentration. This decrease in aggregation temperature means that aggregation occurs when the particles are partially swollen. Now, at these lower temperatures, the interaction between the micelles and microgels is one of depletion. This results in phase separation occurring before gelation, as the microgels are free to rearrange to some extent over short time scales before full gelation occurs, resulting in these voids.

At the higher concentrations of microgels and micelles, demixed phases were observed. At these concentrations the system forms microgel-rich phases (Figure 5, blue). At lower microgel and micelle concentrations, these droplets remain stable over the time scale of the experiment (light blue), but over long time scales (hours) they eventually coalesce leading to macroscopic phase separation (Figure S4). With increasing polymer concentration the rate of droplet coalescence increases, and phase separation proceeds (Figure 5, dark blue) over the time frame of the experiment (Figures S5 and S6).

At higher microgel concentrations, lower micelle concentrations are needed to induce phase separation. At micelle concentrations above ∼7 wt %, the aggregation temperature becomes strongly dependent on microgel concentration and drops rapidly (Figure 4). Phase separation (droplets coalescing) was found to occur when the aggregation temperature was lowest (below 31 °C), as aggregation in these samples occurs over the largest time/temperature range. Videos showing the time evolution of each phase are included in the Supporting Information.

### Mechanistic Description of Phase Behavior

The precise mechanism at work for the complex behavior we observe in mixtures of pNIPAM and triblock copolymer is yet to be fully understood, and in this paper we provide a speculative discussion about the underlying phenomena, based on our experimental data set. The precise mechanism for gel formation is a topic of further research. Here we present our interpretation of the differences in behavior that we observe between fast and slow heating rates. We shall interpret this in terms of the effective interaction potential between the microgels, which we now seek to describe. We describe three temperature regimes in which the behavior of the two components of the system changes, underlying the temperature-dependent phase behavior that we see.

#### Low-Temperature Regime

**Figure 6:**
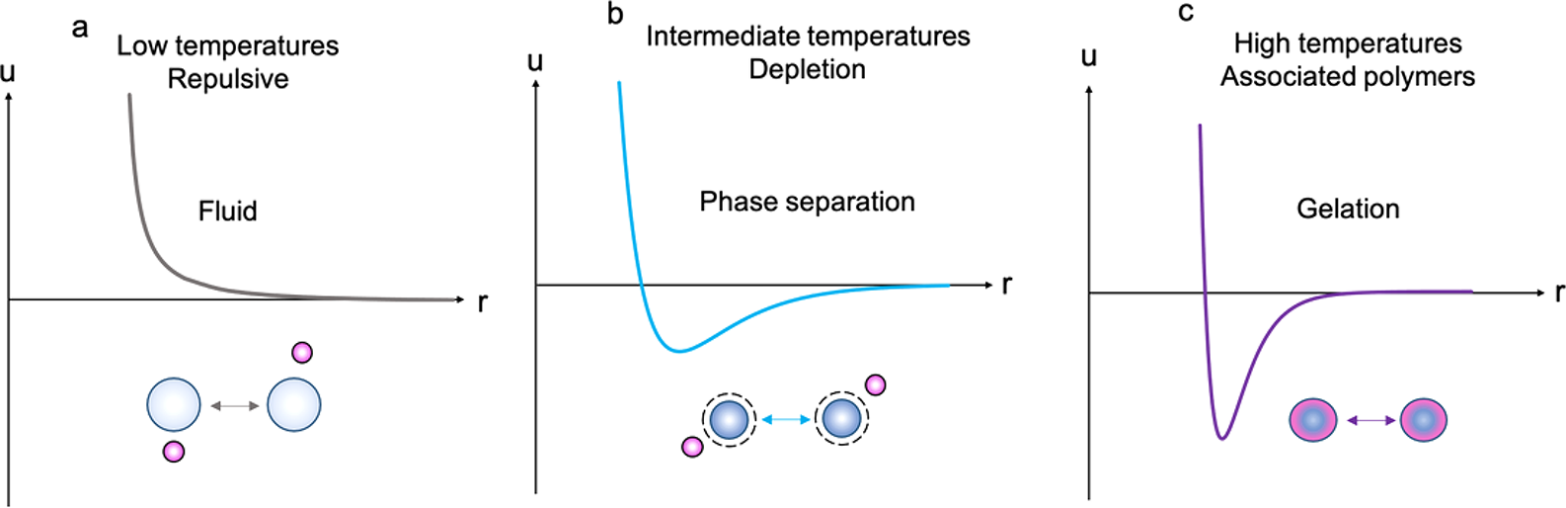
Schematic of the interaction potentials between microgel particles at different temperatures in the presence of triblock copolymer micelles. At low temperatures (a), the interaction between microgels is purely repulsive (fluid). At intermediate temperatures (b), weak vdW interactions now dominate and a depletion force is also present due to the nonadsorbing micelles, which results in liquid phases rich in the two polymer species (phase separation). At high temperatures (c), pNIPAM microgels now associate with the micelles, resulting in the microgels not fully collapsing. There are now strong vdW forces between them, and the particles stick together as a result of association (leading to gelation). The blue circles represent the pNIPAM microgels at different extents of deswelling.

At low temperatures, around room temperature, fully swollen microgels are soft colloidal particles with a layer of extended linear polymer chains. These swollen particles are purely repulsive and vdW interactions are very small, due to the highly diffuse nature of the outer regions of the particles (Figure 6a).^42^

#### Intermediate-Temperature Regime

At intermediate temperatures around *T*_VPT_ when the microgels are partially deswollen, the effective interaction potential between them becomes increasingly attractive. Partly, this is due to vdW attractions between the microgels which start to appear close to the *T*_VPT_. However, due to the microgels still being partially swollen at these temperatures, the vdW potential well is relatively shallow and the interaction remains relatively soft (Figure 6b). Therefore, phase separation at these temperatures is likely driven by depletion, where the nonadsorbing micelles cause aggregation. This depletion interaction results in droplet formation and after sufficient time, phase separation. Because the strength of the depletion interaction depends on the micelle concentration, it is only at the highest concentration of depleting micelles that we see depletion-driven phase separation. Furthermore, there was no evidence of association of the micelles with the microgels using DLS (Figure 3), evidenced by similar microgel diameter at these temperatures in the presence of triblock copolymer.^2^

#### High-Temperature Regime

When the microgels are heated to temperatures above *T*_VPT_ (around 35 °C), they collapse and are often modeled as attractive spheres due to the increased vdW interactions between them and their increased hydrophobicity.^42^ These collapsed particles are more likely to aggregate due to these attractions. These colloids are stabilized solely by the residual charge on the particles left from the initiator during synthesis.^50^

In this high-temperature range, if droplets have already formed due to phase separation at intermediate temperature, the system remains demixed at increased temperature; however, the droplets undergo coarsening (Figure S6), indicating an arrest inside the droplets due to the interactions between the microgels being sufficiently strong at the high concentration densities. On the other hand, if no depletion-induced phase separation took place at intermediate temperature due to either the rate of temperature increase being too fast or the micelle concentration being insufficient, the microgels and the micelles then associate at this higher temperature. Upon collapse, the microgels are harder, and the vdW forces are stronger, corresponding to a deeper attractive potential well (Figure 6c). This results in the particles being unable to rearrange once association occurs, as they are too “sticky”, leading to gelation. Figure 1 (lower scheme) indicates how the interaction potentials relate to the structures observed in the case that aggregation is driven by vdW attractions between collapsed microgels.

This difference in behavior with heating rate indicates that the equilibrium phase is not accessed when the samples are heated quickly (10 °C min^–1^), resulting in gelation. In this regime, the microgels and the micelles associate very quickly as the temperature increases rapidly above that at which the system is dominated by microgel–micelle attractions, and the collapse of the microgels also occurs over a fast time scale. This results in the depletion effects at intermediate temperatures being bypassed, resulting in gel network formation (Figure 1, top scheme). Because of the strong vdW forces between the particles, once the network has formed, there is little ability for particles to rearrange. Therefore, the kinetically favored state forms: collapsing gel networks that undergo syneresis.

### Salt-Induced Gels

**Figure 7:**
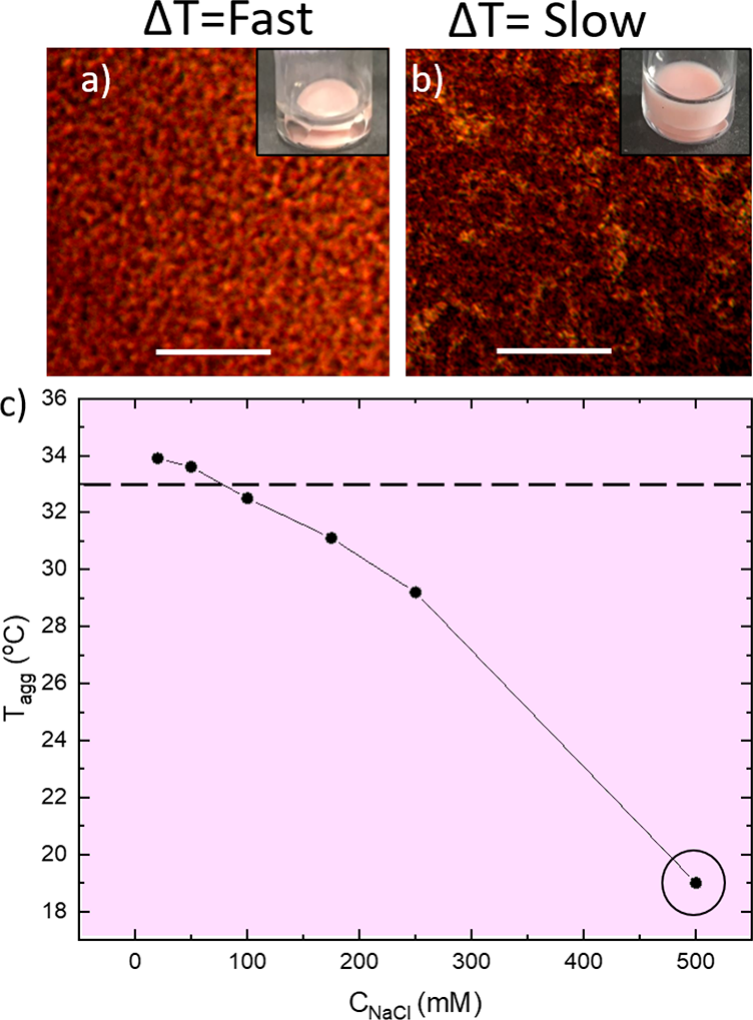
Figure highlighting the effect of heating rate on pNIPAM gels in the presence of salt. DIC images of pNIPAM samples heated quickly (a) and heated slowly (b), with their equivalent macroscopic samples as insets. The samples were at 4 wt % pNIPAM and 500 mM NaCl, highlighted with a black circle in panel c. All images were taken at 35 °C. (c) is the temperature at which aggregation is first observed on the DIC microscope; all samples contain 4 wt % pNIPAM. The dashed line indicates the temperature below which phase separation is observed for mixtures of pNIPAM and triblock copolymer. The scale bar 40 μm. All samples of salt and pNIPAM formed gels regardless of heating rate and gelation temperature.

We now consider the response of the system to the substituting of micelles with salt. pNIPAM is known to also form gels at elevated temperatures in the presence of NaCl; however, when these samples were heated at different rates, there appeared to be no obvious change in structure. Salt is known in the literature to reduce the *T*_VPT_ of pNIPAM microgels,^56^ which is observed in our system, and the decrease in *T*_VPT_ correlates to a decrease in aggregation temperature (Figure 7).

However, little effect was observed in the gels formed in the presence of salt, regardless of gelation temperature and heating rate. This is in contrast to the case without salt, but with micelles, where we find phase separation rather than gelation in the case of slow heating. This highlights the difference in the interaction of the pNIPAM microgels with salt and triblock copolymer micelles. We rationalize this as follows. The presence of salt does lower the *T*_VPT_ of the microgels, which therefore results in collapsing gels forming at all concentrations and heating rates, as the attraction between the microgels becomes strong at lower temperatures and the regime of depletion interaction illustrated in Figure 6b is avoided.

## Conclusions

We have identified a new mechanism of phase separation in a colloidal model system displaying tunable, controllable gelation. This builds significantly on the current understanding of the phase behavior the pNIPAM microgel, PEO–PPO–PEO triblock copolymer system, in which the former form micelles, leading to a micelle–microgel mixture. In particular, our study reveals a previously unreported phase separation to a phase rich in microgels coexisting with a rich in micelles and poor in microgels. While previous work^2^ looked at rapid temperature changes, here we have explored the effect of heating rate, which, along with the composition, has revealed a surprisingly wide range of structures formed in this nonequilibrium system. We provide evidence that demixing in our system at low temperatures is driven by depletion interactions, which are negligible at room temperature but become apparent with increased temperature due to the increased ability of the micelles to be depleted at higher temperatures. At high temperatures, an associative mechanism of gelation dominates, as hydrophobic interactions and hydrogen bonding cause the microgels and micelles to associate. In this way, the microgel–micelle system exhibits two competing mechanisms for demixing. We can control which is selected with the heating rate.

These mixtures form temperature-responsive gels upon heating, and we have shown that the structure and phase behavior of these systems can be controlled by altering the concentration of the components and by altering the heating rate used for gelation. We highlight the broad range of states accessible for mixtures of pNIPAM microgels and triblock copolymer micelles. In particular, upon high rates of heating, phase separation is suppressed due to gelation and subsequent kinetic arrest. At lower rates of heating, the system spends sufficient time at temperatures where depletion dominates, and the attractions between the microgels are milder; in the absence of kinetic arrest, a more complete demixing occurs, with the formation of liquid-like droplets of microgels. This is reminiscent of the coupling between the rate of change of attraction in “sticky spheres” and the degree of self-assembly.^24^

In addition to its response to the heating rate, our system can be tuned by changing the composition. High concentrations of microgel particles lead to uniform gels, while lower concentrations result in gels with voids. Increasing the concentration of micelles promotes demixing to droplets of microgels and micelles. At higher micelle concentration still, we encounter full phase separation to a phase rich in microgels in coexistence with a microgel poor phase. The temperature at which the microgels demix is dependent on the concentration of both the microgels and the micelles, and at high concentrations demixing occurs when microgels are still in the swollen state.

Our study highlights how studying the phase behavior of a microgel–micelle system can ultimately improve our understanding of the behavior of assembly in microgel–surfactant mixtures and the complex interplay between micellization and phase separation kinetics and, for example, can allow us to access both the equilibrium phase behavior and nonequilibrium gel and droplet structures. Furthermore, we have demonstrated that this is a well-parametrized model system to explore multiple kinetic pathways of self-assembly. Finally, these systems may find use as novel temperature-responsive materials.

## Supporting Information Available

DIC image processing, DLS of the pNIPAM microgels, phase diagram in terms of volume fraction of microgels, DIC images of the droplet phase coalescing over time, droplet size evolution over time, DIC images of droplet phase size evolution (PDF)
Video S1 (MP4)
Video S2 (MP4)
Video S3 (MP4)
Video S4 (MP4)
