# Supplementary material for: Controlling Kinetic Pathways in Demixing Microgel–Micelle Mixtures
Source: Langmuir. 2023 Jan 9;39(3):1010–8. doi: 10.1021/acs.langmuir.2c02583 (PMC9878723; doi:10.1021/acs.langmuir.2c02583)
Supplement: Supplementary file 1 — la2c02583_si_005.pdf [file la2c02583_si_005.pdf]

Electronic Supplementary Information

Controlling kinetic pathways in demixing microgel-micelle mixtures

S L Fussell,[*,†,‡] C P Royall,[†,¶,§,‡] and J S van Duijneveldt[†]

†*School of Chemistry, University of Bristol, Cantock's Close, Bristol, BS8 1TS, UK*

‡*Bristol Centre for Functional Nanomaterials, University of Bristol, Tyndall Avenue, Bristol, BS8 1TL, UK*

¶*Gulliver UMR CNRS 7083, ESPCI Paris, Université PSL, 75005 Paris, France.*

§*HH Wills Physics Laboratory, University of Bristol, Tyndall Avenue, Bristol, BS8 1TL, UK*

E-mail: sian.fussell@gmail.com

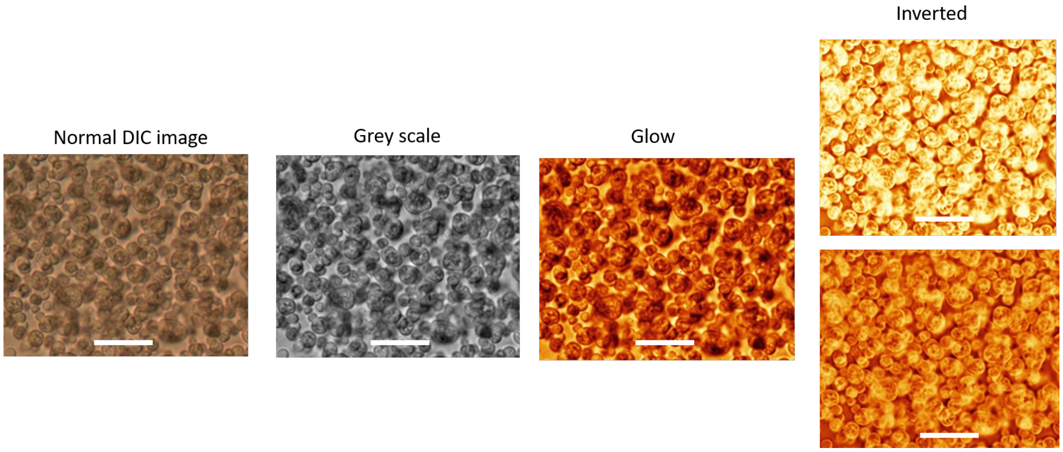

Figure S1: Figure highlighting the editing process used to highlight the regions rich in polymer in images taken using the DIC microscope. DIC images are converted to grey scale, then converted to glow, then the images inverted so that the polymer rich regions appear bright and the polymer poor regions appear dark.The scale bar is 40 μm.

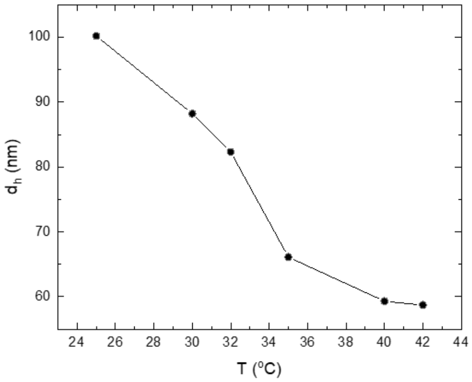

Figure S2: Hydrodynamic diameter of the pNIPAM microgels as a function of temperature.

Figure S1 highlights how differential interference contrast (DIC) images were edited in this work. The images were converted to grey scale, then converted to the glow colour palette, then the image inverted. The brightness of the image was then adjusted. This results in the polymer rich regions appearing bright and the polymer poor regions appearing dark.

Figure S2 contains the deswelling data for the pNIPAM microgels used in this study, determined using dynamic light scattering.

Figure S3 highlights the phase behaviour of pNIPAM microgel in the presence of triblock-copolymers as a function of pNIPAM volume fraction. The volume fraction of the microgels

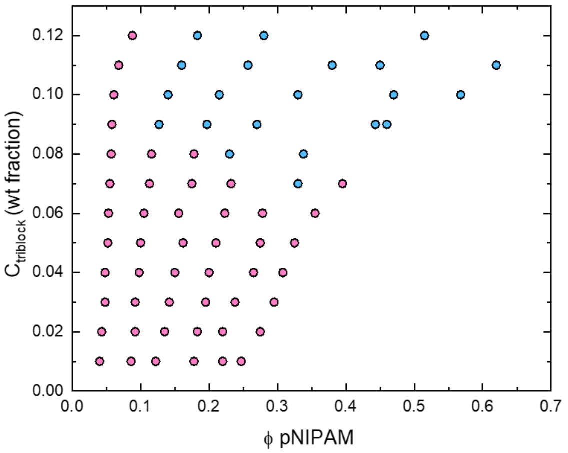

Figure S3: Figure highlighting the phase behaviour pNIPAM microgels in the presence of triblock-copolymer as a function of volume fraction. The pink data point indicate samples that form gels, the blue data points indicate the samples that phase separate. Each data point is at a different temperature as the volume fraction of the microgels is temperature dependent.

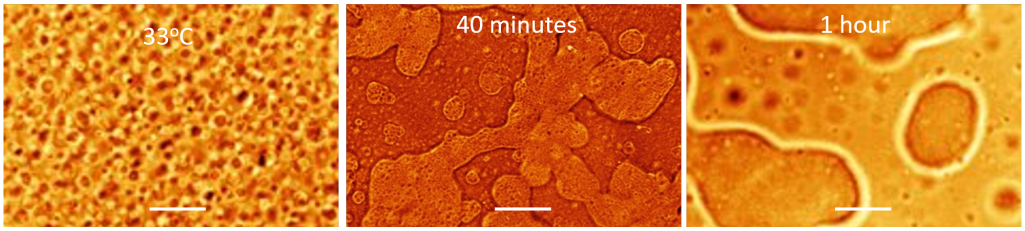

Figure S4: Images of droplets coalescing over time. The concentration of the sample is 4 wt% pNIPAM and 8 wt% triblock-copolymer. The scale bar is 40 μm.

is temperature dependant, as the microgels collapse at increased temperature. Therefore, all the data points are at taken at different temperatures. Each data point is the lowest temperature where aggregates were observed, represented as volume fraction rather than temperature as in Fig 4 in the main text. At lower volume fractions of pNIPAM gels form, indicated in pink. At higher volumes fractions of pNIPAM, phase separation occurs, indicated in blue. The volume fraction has been estimated from the volume fraction of water inside the microgels and the diameter and various temperatures.[?]

Figure S4 highlights the evolution of the droplet phase over time. Droplets begin to form upon heating, then when held at 33°C, the droplets continue to grow and eventually merge. This results in phase separation at prolonged times.

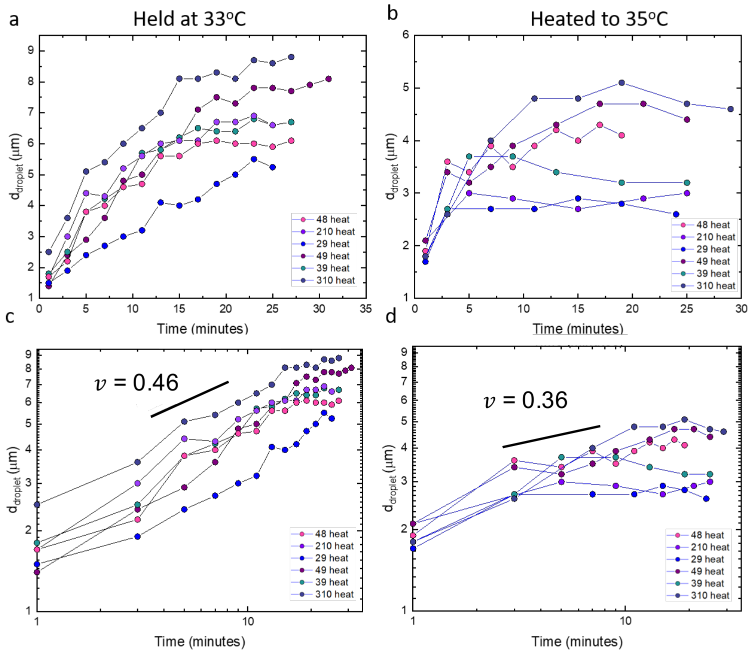

Figure S5: Summary of the droplet size evolution over time, for the concentrations where droplets were indicated to form in figure 1. Droplet size evolution over time when the samples are heated at a rate of 0.1 °C/min to 33°C (a) or 35°C (b). The same data has been plotted on a log time scale in (c) and (d). The time is started from the first indication of aggregates forming. 4 wt% pNIPAM, 9 wt% triblock (maroon), 4 wt% pNIPAM, 8 wt% triblock (pink), 3 wt% pNIPAM, 10 wt% triblock (grey), 3 wt% pNIPAM, 9 wt% triblock (green), 2 wt% pNIPAM, 10 wt% triblock (purple), 2 wt% pNIPAM, 9 wt% triblock (blue) The black lines are the average calculated fitted power laws for the droplet growth series at both temperatures for all droplet sizes in the series.

Figure S5 shows the evolution of droplet size for the series of samples that form droplets. There is a general increase of droplet size with time, tending to a plateau value at late time. It can be seen that droplets grow to larger sizes when held at 33°C (a), rather than heated to 35°C and held at that temperature. It was also observed that a power law can be used to describe the data set, where samples heated to 33°C scale with gradient $\mu = 0.46$, whereas samples heated to 35°C scale with $\mu = 0.36$.

Figure S6 illustrates how the size of the droplets increases with time.

Figure S7 contains the rheological profile for 5 wt% pNIPAM, 3 wt% triblock-copolymer. In the samples that undergo syneresis, the sample pulls away from the plate resulting in the rheological profile looking similar to pure water rather than a hydrogel. It is clear that the

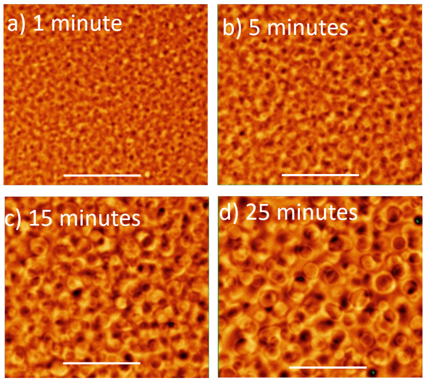

Figure S6: a-d) Optical micrographs of 3 wt% pNIPAM, 9 wt% triblock-copolymer heated to 33°C at a rate of 0.1 °C/min and held at that temperature. The time was started from the first indication of gelation. The scale bar is 10 μm

.

full profile of the pNIPAM samples cannot be accessed therefore this technique is not an accurate method to compare the structures produced in this work.

Table S1: Summary of the pNIPAM particle volume fractions at different concentrations and temperatures

Temperature	3 wt%	4 wt%	5 wt%
25°C	0.34	0.45	0.68
30°C	0.27	0.36	0.45
35°C	0.10	0.14	0.17
40°C	0.07	0.10	0.12

Table S1 includes the volume fractions calculated for the pNIPAM microgels at different wt%.

Videos are also included in the supplementary information to show the formation of the different phases observed in this work.

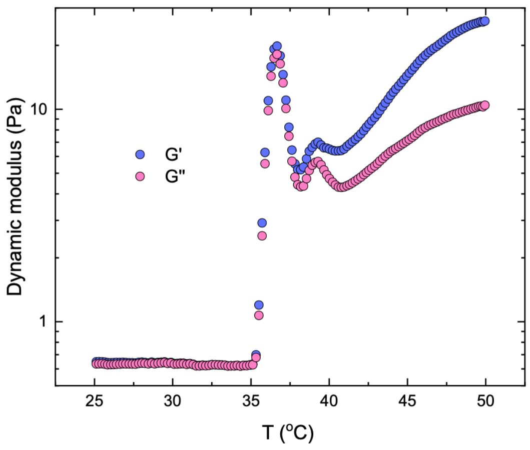

Figure S7: Temperature ramp rheology profile for a sample of pNIPAM (5 wt%) and triblock-copolymer (3 wt%). The strain used was 1% and the frequency was 1 Hz
